# Method for extracting the surface impedance of a generic reflective metasurface

**DOI:** 10.1038/s41598-024-76671-9

**Published:** 2024-11-07

**Authors:** J. G. Smith, I. R. Hooper, N. Clow, A. P. Hibbins, S. A. R. Horsley

**Affiliations:** 1 Department of Physics and Astronomy, Centre for Metamaterial Research and Innovation, University of Exeter, Stocker Road, Exeter, EX4 4QL UK; 2 DSTL, Porton Down Salisbury, Wiltshire, SP4 0JQ UK

**Keywords:** Characterization and analytical techniques, Applied physics, Electronics, photonics and device physics, Electrical and electronic engineering

## Abstract

We develop a method for the extraction of the surface impedance tensor of a generic reflective metasurface using an analytic relation between the tensorial surface impedance and the four polarisation-dependent reflection coefficients. We apply this technique to experimental data obtained from a metasurface with a rhomboidal unit cell in the 16–26 GHz range, but note that it could be applied to reflective metasurfaces in any frequency regime. The extraction method can also be applied to model data to facilitate the design process of spatially graded tensorial metasurfaces that allow for full control of the form of the scattered field.

## Introduction

Through structuring on a sub-wavelength scale, metamaterials gain tailored effective properties—e.g. refractive index and impedance—that determine wave propagation ^1^. Metasurfaces are the planar case, where sub-wavelength elements are arranged in a two-dimensional array ^2–6^, and have been explored for applications^7^ such as anomalous reflection/refraction^8–11^, including reflectionless Huygens metasurfaces^12^, wavefront shaping/beamforming^13–16^, wave guidance/radiation control^17^, and polarisation conversion^16,18–23^. It is known that the local environment around a radiating element affects how much power is emitted via the Purcell effect^24,25^ due to a modification of the local density of states^26^. Hence, metasurfaces are also often used in antenna applications to manipulate both the emitted power and radiation pattern through the local control of the surface impedance^27^, including the development of so-called artificial magnetic conductors for low-profile, high-gain, antennas ^28,29^.

For metasurfaces the usual constitutive properties one might use for bulk materials, such as permittivity and permeability, or the bulk impedance, no longer have physical meaning. Instead, the surface impedance can be used as a homogenised effective material property that will determine the magnitude, phase, and polarisation state of waves reflected from the surface. The use of a surface impedance to describe the properties of interfaces has a long history, certainly going back to the early 60s and the work of Senior^30,31^ and, following the onset of research into metasurfaces starting in the late 1990s, it continues to be the standard means of describing the properties of metasurfaces. The use of such homogenised constitutive properties is essential when designing more complex metasurfaces—full-wave modelling of a graded metasurface (such as a planar lens, for example) is oftentimes impossible due to the need to include the microscopic details of the metasurface in any calculation. However, modelling the required spatial surface impedance profile needed to give a particular modality can be relatively straightforward^10,32–35^. As such, a standard design process is to run relatively small (computationally) models of individual unit cells with periodic boundary conditions that enables the design of metasurface elements that will *locally* produce the required surface impedance.

It is a relatively simple matter to extract the surface impedance from an isotropic scalar metasurface from its reflectivity (whether measured experimentally or via modelling). However, metasurfaces with isotropic scalar impedances do not allow one to have full control over the scattered field (direction, phase, and polarisation). For full control one requires a tensorial surface impedance (TSI), which offers a degree of control over the surface currents ^17,32,36^ that is impossible with a scalar impedance, allowing for the arbitrary transformation of both the field polarisation  ^37,38^, and surface power flow ^39–42^. Such generalized impedance metasurfaces have applications in the design of modified ground planes for antenna applications ^43^, waveguides ^44,45^, polarisation converters ^46^, and leaky wave antennas ^34,35,47–51^. It is clear that a means for extracting the TSI of a metasurface design would be desirable for designing graded metasurfaces, whilst from a metrology perspective a simple means of characterising real-world surfaces would also have significant merit.

Within the literature there are currently two main routes to extracting tensorial surface impedances. Firstly, it has been shown that they can be calculated from the integrated in-plane fields upon irradiation of the surface with two normal incidence orthogonal polarisations^32,52^, and this was confirmed by comparison to model data. Whilst a useful technique for extracting the TSI from model data, it would be exceptionally difficult to use this technique to experimentally characterise real-world metasurfaces.

The second approach is to extract the tensorial surface impedance from reflectivity measurements as demonstrated by Boregese et al.^53^, in which they require normal incidence illumination and cross- and co-polarised measurements for two orthogonal incident polarisations. However, once again, whilst useful for extracting the TSI from model data, experimentally obtaining good-quality reflectivity data for normal incidence illumination is inherently difficult.

In this paper, a simple method is proposed for extracting the tensorial surface impedance of reflective metasurfaces from reflectivity data obtained at arbitrary angles of incidence. The method is demonstrated on experimentally obtained reflectivity data in the 16-26 GHz range from a metasurface with a rhomboidal unit cell. The same extraction method can be applied to model data, where it could be used as a design tool for spatially graded metasurfaces that allow arbitrary field transformations.

## Extracting the tensorial surface impedance

The surface impedance, *Z*, of a scalar metasurface is given by the ratio of the tangential electric and magnetic fields, $\vec {E}_{\parallel }=Z(\hat{n}\times \vec {H}_{\parallel })$, where $\vec {E}_{\parallel }$ and $\vec {H}_{\parallel }$ are the in-plane electric and magnetic fields, and $\hat{n}$ is the surface normal. In the scalar case there is no mixing of the polarisations, but more generally, via symmetry breaking, such mixing is possible and the surface impedance must instead be described by a tensor  ^54^,

1 $$\begin{aligned} \begin{bmatrix} E_x\\ E_y\\ \end{bmatrix} = \begin{bmatrix} Z_{xx} & Z_{xy}\\ Z_{yx} & Z_{yy}\\ \end{bmatrix} \begin{bmatrix} -H_{y}\\ H_{x}\\ \end{bmatrix} \end{aligned}$$

where $E_{i}$ and $H_{i}$ are the in-plane electric and magnetic field components, and the surface normal is along $\hat{z}$. In the scalar case - where $\underline{\underline{Z}}$ is diagonal - the *x* component of the electric field is proportional to the *y* component of the magnetic field (and vice versa), whereas in the fully tensorial case, all field components are linked.

**Figure 1:**
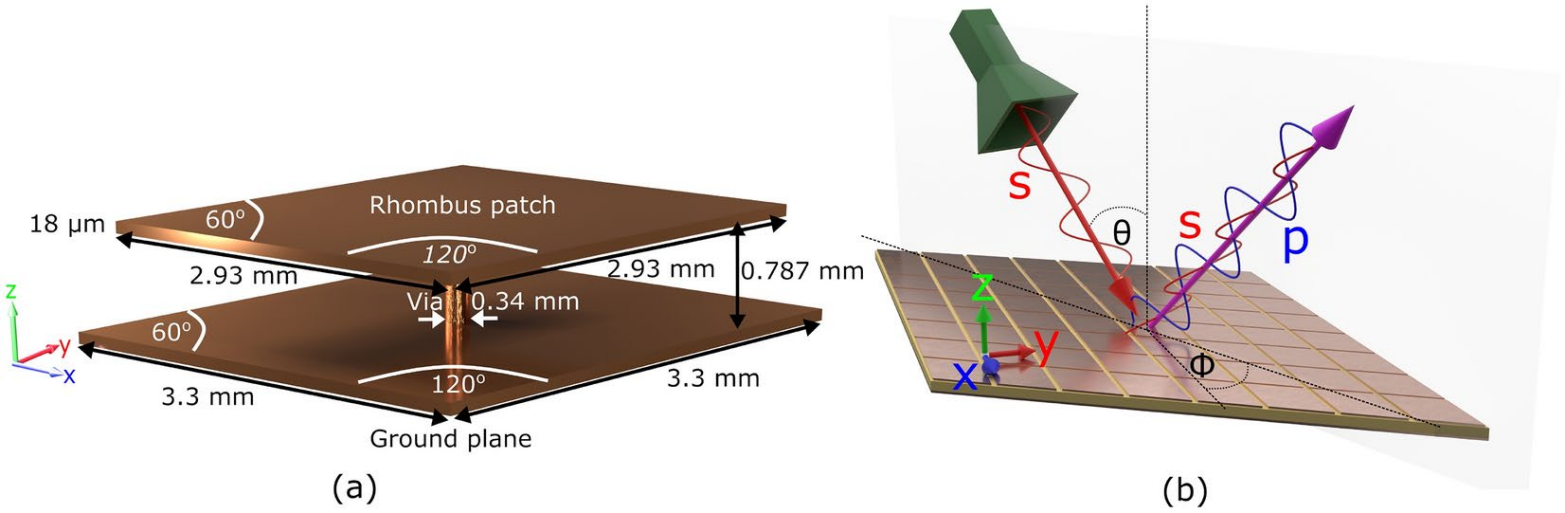
(**a**) Metasurface unit cell. A rhombus shaped metallic patch (side lengths 2.93 mm) is connected by a conducting via (length 0.787 $\textrm{mm}$) to a ground plane. The interior volume below the patch is filled with Nelco-9220 dielectric, which has a dielectric constant of $\epsilon _r=2.21+0.006 \textrm{i}$ (corresponding to a loss tangent of $\tan (\delta )=0.00271$)^55^. (**b**) Tensorial impedance metasurface: Such a surface exhibits a tensorial surface impedance, and can convert between polarisations. As an example here an s-polarised ($\vec {E}$ field perpendicular to plane of incidence) plane wave is partially converted into both s- and p-polarised waves. $\theta$ and $\phi$ are the polar and azimuthal incident angles respectivley.

The reflection coefficients for a tensorial impedance surface can be generally derived from Eq. (1), which can be inverted to give the surface impedance tensor as a function of the four co- and cross-polarised reflection coefficients (see Appendix 1 for the full derivation),

2 $$\begin{aligned} Z_{xx}&=\frac{(r_{\text {ps}}r_{\text {sp}}+(1-r_{\text {ss}})(1-r_{\text {pp}}))\cos {(\theta )}}{(1+r_{\text {pp}})(1-r_{\text {ss}})-r_{\text {ps}}r_{\text {sp}}},\end{aligned}$$

3 $$\begin{aligned} Z_{xy}&=\frac{2r_{\text {ps}}}{(1+r_{\text {pp}})(1-r_{\text {ss}})-r_{\text {ps}}r_{\text {sp}}},\end{aligned}$$

4 $$\begin{aligned} Z_{yx}&=\frac{2r_{\text {sp}}}{(1+r_{\text {pp}})(1-r_{\text {ss}})-r_{\text {ps}}r_{\text {sp}}},\end{aligned}$$

5 $$\begin{aligned} Z_{yy}&=\frac{r_{\text {ps}}r_{\text {sp}}+(1+r_{\text {ss}})(1+r_{\text {pp}})}{((1+r_{\text {pp}})(1-r_{\text {ss}})-r_{\text {ps}}r_{\text {sp}})\cos {(\theta )}}. \end{aligned}$$

Here $\theta$ is the incident angle (from the normal) and the reflection coefficient subscripts “s” and “p” refer to the polarisation, with the first index indicating the outgoing polarisation and the second index the incoming polarisation. For example, the field generated by an incident *s*-polarised wave (see Fig. 1b) is given by,

6 $$\begin{aligned} E=E_0 e^{ik_x x} \left[ (e^{-ik_z z}+r_{\text {ss}} e^{ik_z z})\hat{y}+r_{\text {ps}}e^{ik_z z} \left( \frac{k_z\hat{x}-k_x\hat{z}}{k_0} \right) \right] e^{-i\omega t}. \end{aligned}$$

To test the validity of Eqs. (2)–(5), a custom tensorial impedance boundary condition^56^ was incorporated into the RF module in COMSOL Multiphysics^57^. A series of randomly generated surface impedance tensors were generated, and the calculated reflectivities from these boundaries were subsequently input into Eqns. 2–5 to re-obtain the input tensorial impedance. Differences between the input and calculated impedances were minimimal, and within the errors expected for such approximate numerical models—see Appendix 2 for details.

Having tested the extraction method on model data, we now turn to an experimental verification using a metasurface with a rhomboidal unit cell and formed of metallic patches separated from a ground plane by a dielectric layer, with vias connecting the patches to the ground plane. The geometry of the sample is shown in Fig. 1a. This sample was chosen due to its known complex reflectivity spectrum that includes polarisation mixing—for previous work on the behaviour of surface waves on this metasurface see reference^58^.

To determine the surface impedance of the metasurface, all 4 polarisation dependent reflection coefficients ($r_{\text {pp}}$, $r_{\text {sp}}$, $r_{\text {ps}}$, $r_{\text {ss}}$) have to be determined. These were obtained for the metasurface shown in Fig. 1b for incident angles of $\theta =45^{\circ }$ and $\phi =0^{\circ }$, both experimentally and through finite element modelling ^57^—see “Methods” section.

**Figure 2:**
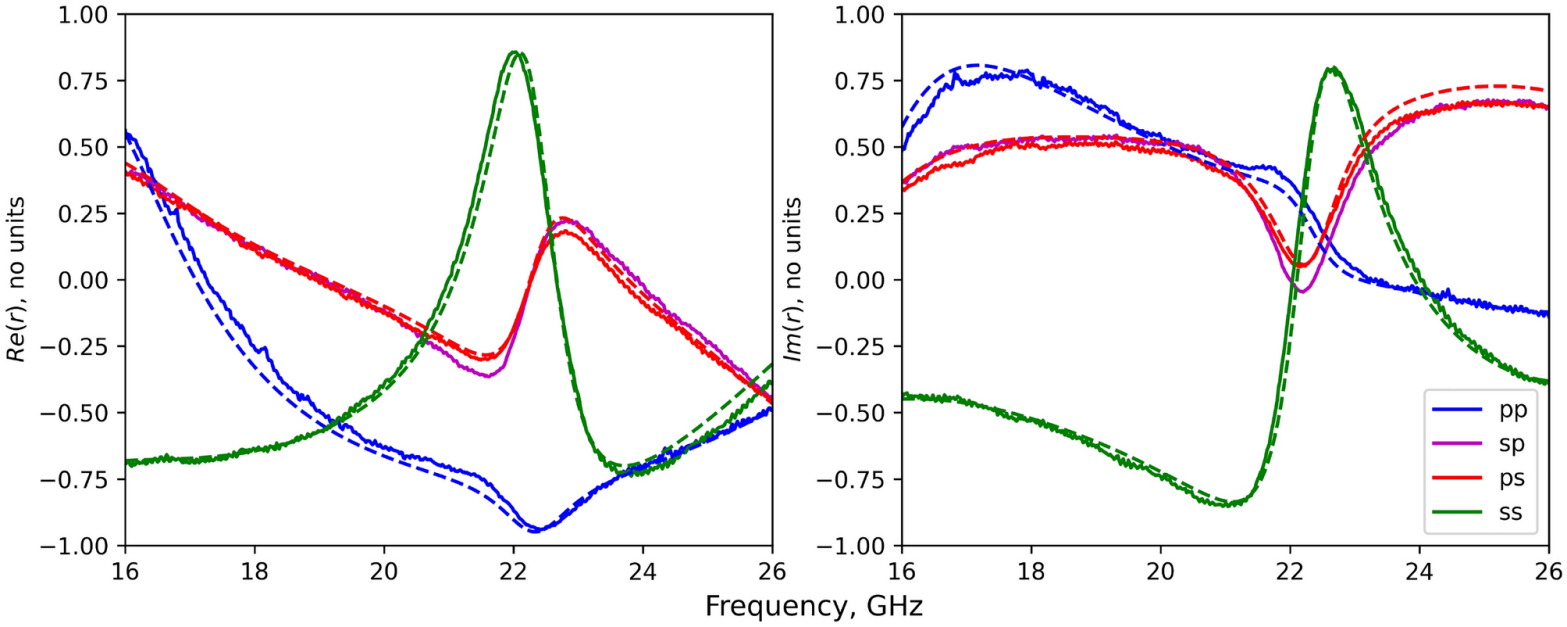
Reflectivity data. The frequency-dependent reflectvity data obtained from the-rhombus patch tensorial-impedance surface depicted in Fig. 1b for $\theta =45^{\circ }$ and $\phi =0^{\circ }$. The solid lines denote experimentally measured reflectivity data using the setup shown in Fig. 4. The dashed lines are model data obtained using COMSOL Multiphysics^57^.

**Figure 3:**
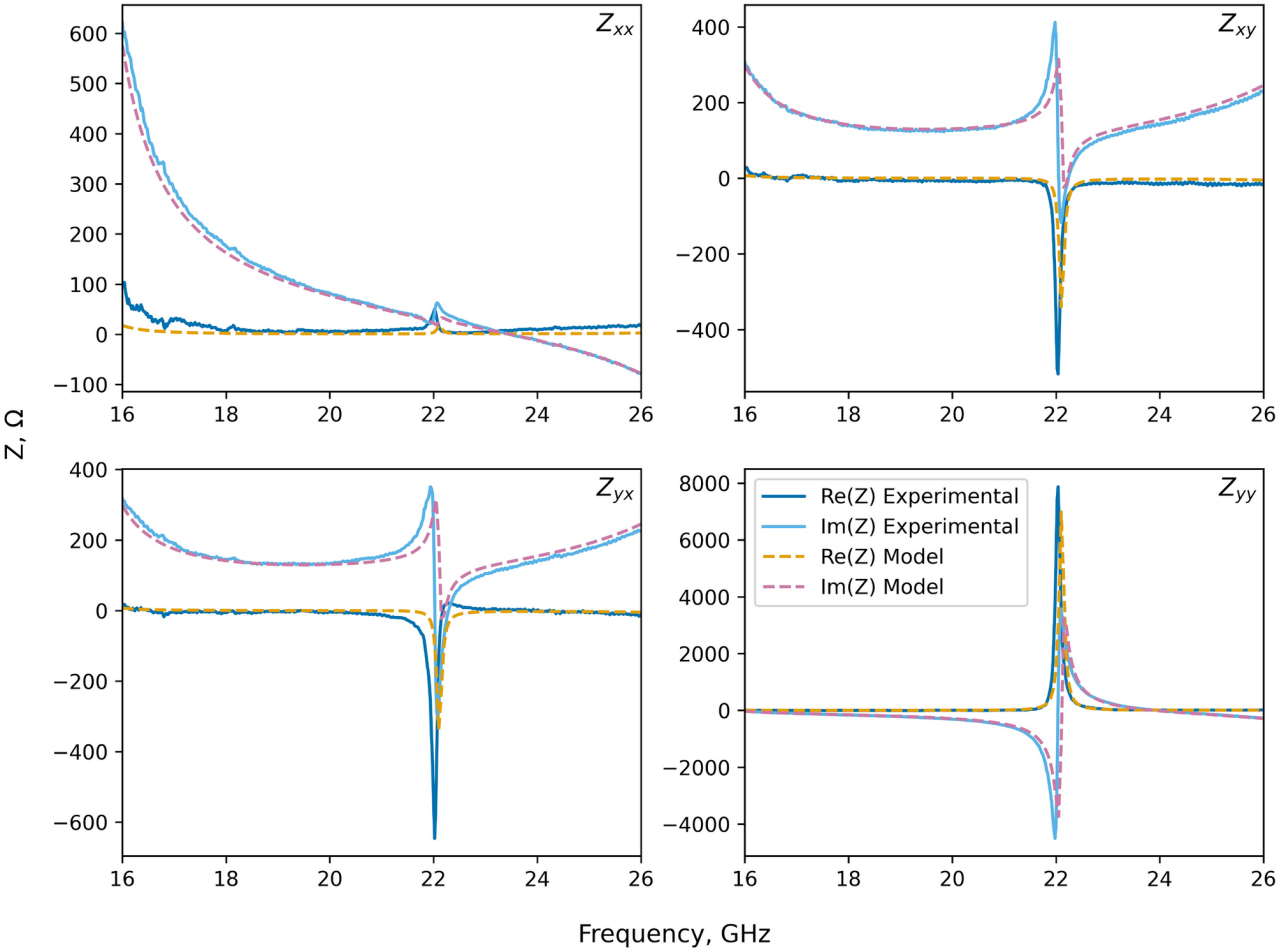
Calculated impedance tensor. The components of the frequency-dependenent surface impedance tensor for the sample depicted in Fig. 1b as calculated using the reflectivity data shown in Fig. 2 and Eqs. (2)–(5).

From the reflectivity data shown in Fig. 2, a resonance is evident in both the unconverted s-polarised reflectivity and the cross-polarised reflectivity at around 22 GHz. When translated into the set of impedance values shown in Fig. 3, they yield a characteristic Lorentzian-type response in the diagonal impedance component $Z_{yy}$, as well as the off-diagonal components $Z_{xy}$ and $Z_{yx}$. The remaining diagonal component of the impedance $Z_{xx}$ also shows a resonant response in this frequency range, although with a much reduced strength.

Note that in Fig. 2 there is a null in the cross-polarisation coefficients $r_{\text {ps}}$ and $r_{\text {sp}}$ since close to resonance the impedance tensor is dominated by the very large value of the diagonal component $Z_{yy}$, meaning that the other components can be treated as approximately zero, thus conserving s- and p-polarisation. Despite the low value in the converted polarisation in this region, there is a rapid change in the phase of the cross-polarisation reflection coefficients with frequency, leading to the observed resonant features in $Z_{xy}$ and $Z_{yx}$. Also note that, unlike the case of a scalar surface impedance, the real part of the off-diagonal component of the impedance tensor does not have a fixed sign and can be negative—whether a surface is dissipative or amplifying is determined by the sign of the real part of the eigenvalues of the impedance tensor, not the individual elements.

At the lower and upper ends of the measured band the impedance values $Z_{xx}$, $Z_{xy}$, and $Z_{yx}$ change rapidly with frequency. These are due to resonances outside the measured band of frequencies associated with different resonant lengths or higher order modes within the metal patch resonators.

Although the experimental results agree very well with finite element modelling, there are some small discrepancies. For example the $Z_{xx}$ component is overestimated by the experiment at the lower end of the frequency band, and the resonant feature at 22.5 GHz is at a slightly lower frequency than in the model, this could be attributed to a frequency dependence in the relative permittivity in the dielectric layer. An overestimate of the loss could arise from the imperfectly flat nature of the sample, causing part of the signal to to scattered away from the detector^59^, an effect that may be increased at lower frequencies due to a broader beam and the corresponding increased region of the sample that is illuminated. Other discrepancies are likely to be a result of small variations from the design dimensions, variation of the dielectric constant from datasheet values, etc.

The above considerations regarding the discrepancies between the model and experimental data are indicators of some of the limitations of this extraction method with real-world samples. The analysis assumes plane-wave illumination, perfectly planar samples, and samples that are uniform over the full size of the irradiating beam. The analysis will not work on graded metasurfaces, curved surfaces, or transmissive surfaces.

## Conclusions

From the expressions for the tensorial impedance of a generic reflective metasurface—written in terms of the 4 polarisation-dependent reflection coefficients—we have developed a method for extracting the tensorial impedance from measured reflectivity data for an arbitrary non-graded metasurface. We have demonstrated the efficacy of this technique, both numerically and experimentally, and have shown that it allows accurate and efficient evaluation of the tensorial surface impedance of a metasurface. In conjunction with finite element modelling the same analysis could be used as a powerful design tool for graded impedance metasurfaces that allow arbitrary field transformations.

## Methods

**Figure 4:**
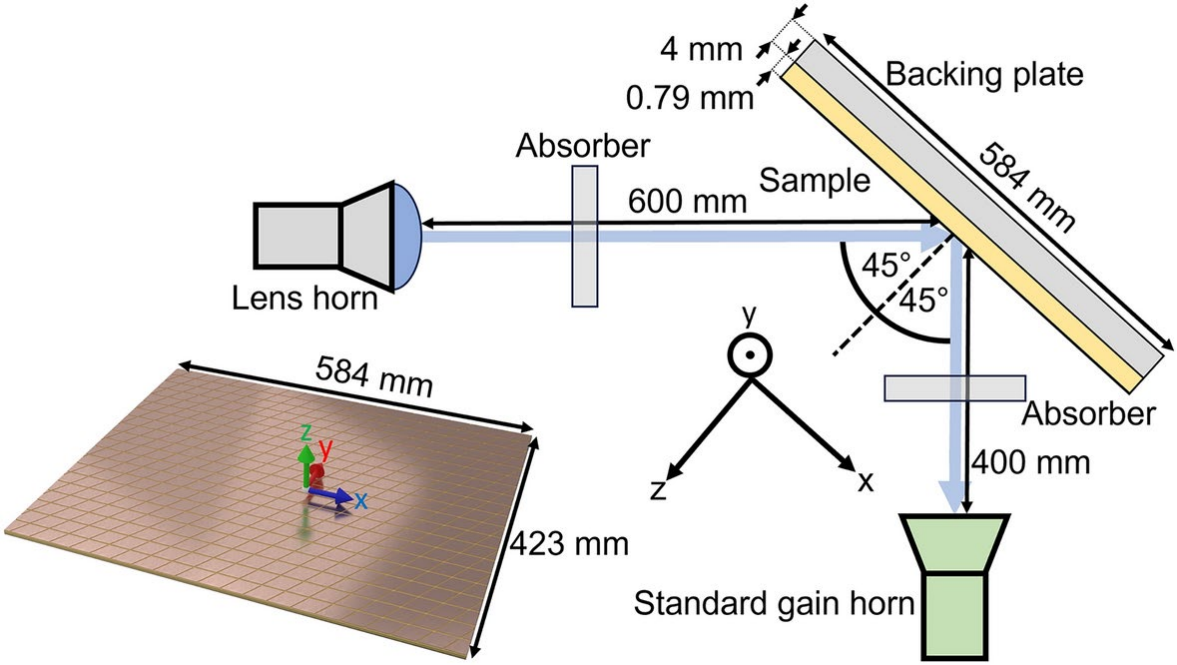
Experimental setup. Schematic of the experimental setup used to measure the 4 reflection coefficients of the tensorial impedance surface. See “Methods” section for details.

In order to determine the tensorial surface impedance, the reflectivity of the sample must be measured for both s- and p- polarisations under separate s- and p-polarised excitation. We did this numerically using finite element modelling (COMSOL Multiphysics with the RF module^57^), where Floquet boundary conditions are applied to the unit cell shown in Fig. 1a. The simulated region consists of the ground plane, dielectric, and patch with a 3.7 cm region of free space above the surface, at the top of which are two ports. One port emits and receives a plane wave of fixed s or p polarisation, with the second port configured to receive the orthogonal polarisation.

The sample consisted of a $584\times 423 \text {mm}$ rectangular sheet of $156\times 147$ rhombic unit cells with dimensions as shown in Fig. 1a. Within the model, the copper parts were described using an impedance boundary condition that accounted for the surface roughness, and therefore the reduced conductivity of the copper from $\sigma =6\times 10^7 \hbox {S m}^{-1}$ to $\sigma =2\times 10^7 \hbox {S m}^{-1}$^60^. Based on the experimental measurements shown in Fig. 2, the relative permittivity of the dielectric filler used in the modelled data of Figs. 2 and 3 is $\epsilon _r=2.21+0.006i$. This was modified slightly from the original data sheet value of $\epsilon _r=2.22+0.002i$ to enable a better match between model an experiment.

Our experiment consisted of a dual horn-antenna setup, as shown in Fig. 4. The reflectivity from the sample as a function of frequency was measured for $\theta =45^{\circ }$ and $\phi =0^{\circ }$, using a Flann microwave lens horn (Model 20810-FA-12224) to provide approximately plane-wave illumination, and a Narda model 638 standard gain horn to receive the reflected radiation. The received signal from the horn antenna was processed using an Anritsu MS4644A Vector Network Analyser. To mitigate standing waves due to reflections from the front faces of the horn antennas, which would have otherwise manifested as rapid oscillations in our measured spectra, partially absorbing foam sheets (ABS-ASF-12 from ABS Technics) were mounted between the horn antennas and the metasurface at a distance of 20 cm from the horns. These essentially detune the standing wave resonances, significantly improving the measured spectra.The sample was adhered to a 4 mm thick aluminium sheet to improve flatness. The reflectivity data from the sample was normalised by the reflectivity of a flat aluminium plate in place of the sample.

## Supplementary Information

Supplementary Information 1.

Supplementary Information 2.

## Data Availability

Data is provided within the manuscript or supplementary information files
