# Supplementary material for: Method for extracting the surface impedance of a generic reflective metasurface
Source: Sci Rep. 2024 Nov 7;14:27141. doi: 10.1038/s41598-024-76671-9 (PMC11544093; doi:10.1038/s41598-024-76671-9)
Supplement: Supplementary file 1 — Supplementary Information 1. [file 41598_2024_76671_MOESM1_ESM.pdf]

Additional information

Appendix 1: Derivation of the Tensor impedance boundary reflection coefficients

We define a 2D system in which a plane-wave propagating through a medium with a (scalar) impedance equal to free space (Z_0), is reflected from a boundary with a general tensorial surface impedance. The surface sits in the $z = 0$ plane with the incident and reflected waves propagating in the $x - z$ plane.

The electric field in the $z > 0$ half-space consists of a $\hat{y}$ component formed of a $\hat{y}$–polarised incident field and a polarisation conserved reflection from the surface. A second reflection term denotes the converted polarisation, which will be polarised orthogonally to both the $\hat{y}$ direction and the direction of propagation $\vec{k}$, giving a total electric field in the upper half-space of

$$E = E_0 e^{ik_x x}\left((e^{-ik_z z} + r_{\mathrm{ss}} e^{ik_z z})\hat{y} + r_{\mathrm{ps}} e^{ik_z z}\left(\frac{k_z\hat{x} - k_x\hat{z}}{k_0}\right)\right). \tag{7}$$

The reflected wave will be travelling in the positive k_x, k_z direction, meaning the p-polarised component will take the form,

$$\hat{y} \times \vec{k}_r = \frac{k_z}{k_0}\hat{x} - \frac{k_x}{k_0}\hat{z}.$$

Calculating the differentials and then applying boundary conditions ($x = y = z = 0$), the boundary is located in the $z = 0$ plane, and a ray incident at (0,0) is equivalent to any other point on the surface. We then calculate $\nabla \vec{\times} E$

$$\vec{\nabla} \times \vec{E} = ik_z E_0(1 - r_{\mathrm{ss}})\hat{x} + ik_x E_0(1 + r_{\mathrm{ss}})\hat{z} + \frac{iE_0}{k_0}(k_x^2 + k_y^2) r_{\mathrm{ps}}\hat{y}.$$

Note that since $k_x^2 + k_y^2 = k_0^2$, and the curl of the electric field is the time derivative of the magnetic field, it follows that,

$$\vec{H} = \frac{E_0}{\omega_0\mu_0}\begin{bmatrix} k_z(1 - r_{\mathrm{ss}}) \\ k_0 r_{\mathrm{ps}} \\ k_x(1 + r_{\mathrm{ss}}) \end{bmatrix} = \frac{E_0}{k_0 Z_0}\begin{bmatrix} k_z(1 - r_{\mathrm{ss}}) \\ k_0 r_{\mathrm{ps}} \\ k_x(1 + r_{\mathrm{ss}}) \end{bmatrix}. \tag{8}$$

where Z_0 is the impedance of free space.

Now, applying the tensor impedance boundary condition (eqn. 1) and inserting the E_x and E_y terms from equation (6), we obtain

$$\begin{bmatrix} \cos(\theta) r_{\mathrm{ps}} \\ 1 + r_{\mathrm{ss}} \end{bmatrix} = \begin{bmatrix} -Z_{xx} r_{\mathrm{ps}} + Z_{xy}\cos(\theta)(1 - r_{\mathrm{ss}}) \\ -Z_{yx} r_{\mathrm{ps}} + Z_{yy}\cos(\theta)(1 - r_{\mathrm{ss}}) \end{bmatrix}. \tag{9}$$

We now have the first two of 4 simultaneous equations that we will need to solve for the impedance in terms of the reflection coefficients. The second two are obtained by considering the case of an incident transverse magnetic field, where the field in the upper half space is defined as,

$$H = H_0 e^{ik_x x}\left(\left(e^{-ik_z z} + r_{pp} e^{ik_z z}\right)\hat{y} + r_{sp} e^{ik_z z}\left(\frac{k_z\hat{x} - k_x\hat{z}}{k_0}\right)\right).$$

Undertaking a similar process to that for the E-field above, one obtains a second pair of equations,

$$\begin{bmatrix} -\cos(\theta)(1 - r_1) \\ -r_{\mathrm{sp}} \end{bmatrix} = \begin{bmatrix} -Z_{xx}(1 + r_{pp}) + Z_{xy}\cos(\theta) r_{sp} \\ -Z_{yx}(1 + r_{pp}) + Z_{yy}\cos(\theta) r_{sp} \end{bmatrix}, \tag{10}$$

and it is then a simple matter of substitution and elimation to obtain equations 2–5

Appendix 2: Equation verification using a custom tensorial impedance boundary condition

In order to test the validity of equations 2-5 we modified the impedance boundary condition in Comsol Multiphysics to allow a tensorial description of the impedance[46]. This tensorial impedance boundary condition (TIBC) was incorporated into a 2D Comsol model consisting of a cuboid domain with periodic boundary conditions on sides facing x and y, ports at the top configured for outputting either s or p–polarisations and receiving both, and the TIBC on the bottom boundary. A set of tensorial impedances was then randomly generated, and the polarisation conserved and converted reflectivities for both s and p incident polarisations were determined. These reflectivities were subsequently used with equations 2-5 to calculate the impedance of the TIBC. The comparison of the inputted and calculated impedances can be seen in figure 5, and shows excellent agreement.

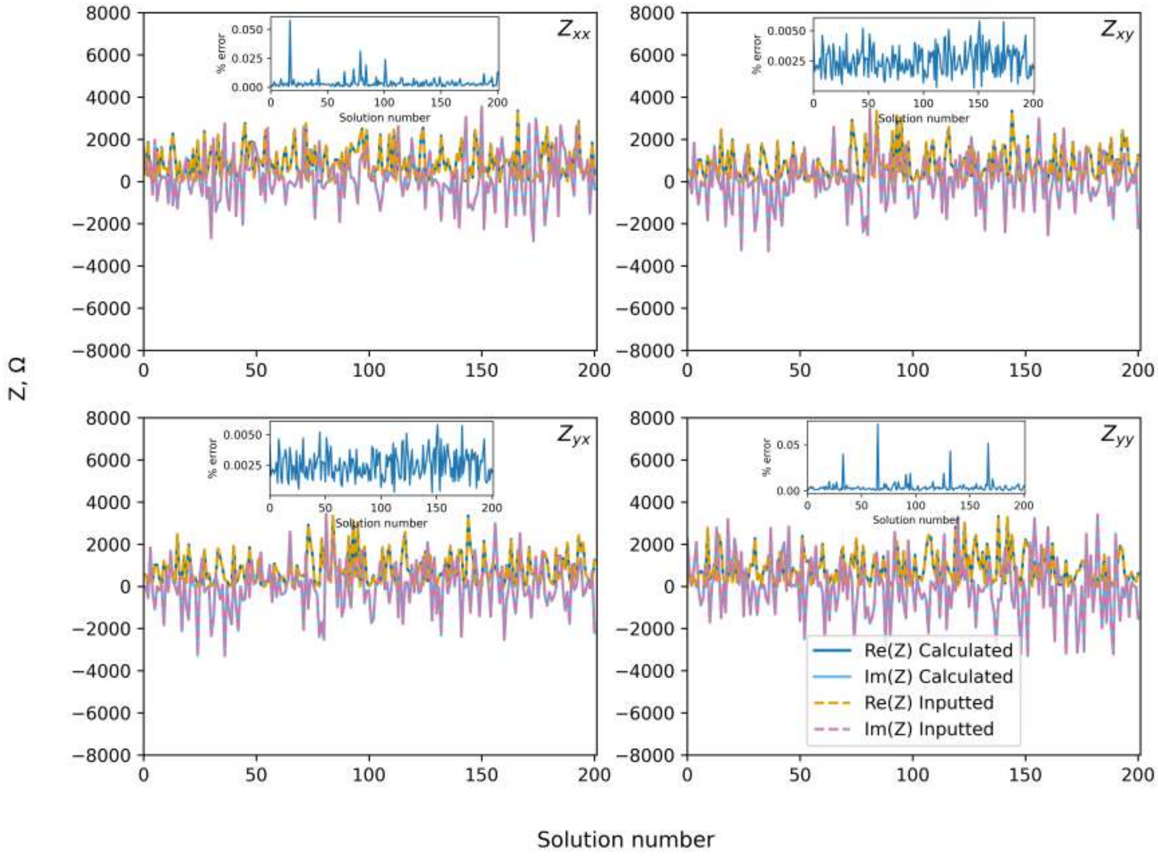

Figure 5. Testing the surface impedance extraction: Comparison of the tensorial surface impedance components input into the COMSOL Multiphysics model with those subsequently extracted from the reflectances from that model using eqns. 2-5.

No Accession codes are applicable

The authors declare no competing interests

The corresponding author is responsible for submitting a competing interests statement on behalf of all authors of the paper. This statement must be included in the submitted article file.
